# The impact of systemic long‐term medications for the development of age‐related macular degeneration

**DOI:** 10.1111/aos.70113

**Published:** 2026-03-06

**Authors:** Hanna Heloterä, Reijo Sund, Toni Rikkonen, Kai Kaarniranta

**Affiliations:** ^1^ Department of Ophthalmology University of Eastern Finland Kuopio Finland; ^2^ Clinical Research Center Kuopio University Hospital Kuopio Finland; ^3^ Kuopio Musculoskeletal Research Unit (KMRU) University of Eastern Finland Kuopio Finland; ^4^ Department of Ophthalmology Kuopio University Hospital Kuopio Finland

**Keywords:** ageing, age‐related macular degeneration, antithrombotic, corticosteroids, immunosuppressants, KFPS, OSTPRE, systemic medication

## Abstract

**Purpose:**

Age‐related macular degeneration (AMD) is a leading cause of central vision loss in the elderly; however, the systemic factors that modulate its incidence and progression remain unclear. We sought to determine whether long‐term use of systemic medications, including diabetes and antithrombotic medications, corticosteroids and immunosuppressants, is associated with the development of AMD.

**Methods:**

The data included a pooled cohort of two follow‐up studies: the Kuopio Osteoporosis Risk Factor and Prevention Study and the Kuopio Fall Prevention Study. A total of 16 518 women born between 1932 and 1946 living in eastern Finland were followed up between 1993 and 2021. The long‐term medication use was indicated as ≥5 years of regular purchase of drugs. Incidence for AMD was analysed per long‐term medication group, but also during the 5 years period in age matched groups.

**Results:**

We observed increased odds for AMD among long‐term users of systemic corticosteroids (HR 1.348, *p* < 0.001), immunosuppressants (HR 1.623, *p* = 0.004) and antithrombotic (HR 1.145, *p* = 0.042) medication. However, results with antithrombotic medications were not as consistent as with corticosteroids and immunosuppressants. Interestingly, the mean age at diagnosis of AMD was later among antithrombotic medication users compared to the control group (77.9 vs. 76.1, respectively, *p* < 0.001) and earlier among patients using other diabetes medications than metformin (75.3 vs. 77.1, *p* = 0.026).

**Conclusions:**

People with long‐term corticosteroid and immunosuppressant use are at greater risk for developing AMD. Further research on systemic components behind corticosteroid and immunosuppressant use, modulating AMD progression, is warranted.

## 1 INTRODUCTION

Age‐related macular degeneration (AMD) is a degenerative condition that affects the macula, the central part of the retina important for sharp and colour vision (Fleckenstein et al., 2024; Vemala et al., 2017). It is diagnosed predominantly in individuals older than 60 years, and the prevalence is increasing most rapidly after 75 years. AMD presents a major cause of blindness worldwide among the aged population (Colijn et al., 2017; Wong et al., 2014). In Western countries, one in seven over 70‐year‐olds and one in three over 80‐year‐olds are at risk of developing AMD. Of these, over one‐third will have severe visual impairment or blindness in both eyes (Colijn et al., 2017; Li et al., 2020; Mitchell et al., 2018). The prevalence of AMD among Caucasians increased from 3.5% in people aged 55–59 years up to 17.6% in those 85 years and older (Colijn et al., 2017). But, the increase in the incidence and prevalence of visual impairment due to AMD in the past decades has shifted to older age in the 2010s when therapies for neovascular AMD (nAMD) became commonly available (Purola et al., 2022). AMD is divided into two main groups: dry AMD (dAMD) and neovascular AMD (nAMD). The average age to get nAMD is approximately at 80 years (Helotera et al., 2024). The incidence and prevalence of nAMD were two times higher in women than in men in three Finnish nationwide health examination surveys (Purola et al., 2025). nAMD is treatable though not curable: regular intravitreal injections of anti‐VEGF (vascular endothelial growth factor) drugs (bevacizumab, ranibizumab, aflibercept, brolucizumab, faricimab) suppress vascular growth, activity and disease progression (Helotera & Kaarniranta, 2022).

In addition to degenerative changes in the deeper layers of the retina (Gurubaran et al., 2025), many environmental factors, metabolic and auto‐immune diseases are associated with AMD (Chen et al., 2014; Helotera et al., 2024; Moir et al., 2023; Velilla et al., 2013; Xu et al., 2020; Zhang et al., 2016). Furthermore, patients with AMD are at increased risk of conditions outside of the eye, such as dementia, Alzheimer's and kidney diseases (Jung et al., 2023; Tsai et al., 2023). As a link to drug use, second‐generation calcium channel blockers and hypertension could be associated with an increased risk for wet AMD development (Loukovaara et al., 2022). There are also some evidence that use of metformin and non‐steroidal anti‐inflammatory drugs may reduce the risk of AMD (Aggarwal et al., 2024; Jiang et al., 2022; Xu et al., 2021; Yang et al., 2024). However, results obtained with aspirin are controversial (Yan et al., 2022). Moreover, it was recently suggested that levodopa would reduce new onset nAMD and may reduce the frequency of needed anti‐VEGF injections during treatment of nAMD (Figueroa et al., 2021; Hyman et al., 2023). Levodopa can cross the blood‐retina barrier and thus observed results can be mediated due to local action of the systemically administered medication (Motz et al., 2020). Interestingly, high systemic levels of IL‐6 are shown to be associated with late AMD and especially with geographical atrophy, whereas low serum levels of TNF‐α are associated with increased visual acuity after anti‐VEGF therapy for nAMD (Khan et al., 2022; Nahavandipour et al., 2020; Rozing et al., 2020). We recently showed that patients on anti‐coagulation medication receive nAMD diagnosis later than patients without medication (Helotera et al., 2024). Previously, Akter et al. showed that anti‐coagulant dabigatran may reduce risk for new nAMD (Akter et al., 2022; Helotera et al., 2024). Thus, the increasing evidence suggests that management of systemic conditions or systemically administered treatments may have a role in the progression of AMD.

In this study, we aimed to explore whether long‐term exposure to systemic medications, including diabetes and anti‐thrombotic medications, systemic corticosteroids and immunosuppressants, is linked to the incidence of AMD.

## 2 MATERIALS AND METHODS

### 2.1 Study population

The study population consisted of a pooled cohort of two follow‐up studies, including the Kuopio Osteoporosis Risk Factor and Prevention (OSTPRE) Study and Kuopio Fall Prevention Study (KFPS), totalling 16 518 women living in the North Savo region, eastern Finland who were followed 1993 onwards. The cohort included Caucasian women born between 1932 and 1946 with a mean baseline age of 54.9 (SD 3.8) years. The OSTPRE and the KFPS studies were approved by the Kuopio University Hospital Ethics Committee in 1989 and 2016, respectively. Both studies have been described in detail previously (Rikkonen et al., 2023; Sund et al., 2014) and were performed according to the ethical standards of the Declaration of Helsinki. Informed consent has been provided before the onset of each data collection.

The analysis utilizes self‐reported health questionnaires for baseline height, weight, smoking, and menopause age. Permissions to register linkages were granted by the Finnish Social and Health Data Permit Authority Findata (Dnro THL/5436/14.06.00/2024, 12.09.2024).

### 2.2 Statistical analysis

The beginning of the year 1993 formed the baseline for the analysis. Depending on the outcome of interest, follow‐up was terminated on the date of death, AMD diagnosis or at the end of the registry period on 31 December 2021. Information on time of death was obtained from the National Causes of Death Register. Women with dAMD were identified with a diagnosis code H35.30 and nAMD patients with a code H35.31 (https://icd‐codes.info/fi/VII/H30‐H36/H35/). AMD cases were identified from national and local health care registers using diagnosis codes (ICD‐10: H35.30 and H35.31, ICD‐9: 3625, ICD‐8: 37710). Based on Finnish guidelines for diagnosis, treatment and follow‐up of nAMD, diagnosis of AMD is carried out by an ophthalmologist specialized in retinal diseases. Diagnosis requires clinical examination including visual acuity (VA) testing, slit lamp microscopy, evaluation of the fundus of both eyes, optical coherence tomography (OCT) and angiography (fluorescein or OCT‐A). In challenging cases, indocyanine green angiography (ICGA) may be used (Tuuminen et al., 2017).

All AMD diagnoses were recorded from the hospital discharge register (HILMO) and statistics on primary health care (AvoHILMO).

The time‐dependent Cox proportional hazards models were employed in survival analysis with their respective 95% CI to account for the impact of long‐term medication on AMD incidence over time. Group means and proportions were compared using an independent samples *t*‐test and Pearson chi‐squared test. Women were classified as long‐term medication users after receiving annual reimbursements for prescription costs over a period of five consecutive years. The medications were identified with Anatomical Therapeutic Chemical (ATC) codes from the Finnish Social Insurance Institution's national drug reimbursement registry (KELA).

The long‐term medications of interest were categorized by using the respective ATC codes for: Diabetes medication (A10) and diabetes medications containing metformin (A10BA02, A10BA01, A10BA03, A10BD02, A10BD03, A10BD05, A10BD07, A10BD08, A10BD10, A10BD1, A10BD13‐18, A10BD20, A10BD22‐23, A10BD25‐27), antithrombotic agents (B01, B01AA, B01AB, B01AC, B01AE, B01AF and B01AX), immunosuppressants (L04) and systemic corticosteroids (H02).

Data were analysed by SPSS, version 29.0.2.0 (SPSS, Chicago, IL, USA).

## 3 RESULTS

### 3.1 Study population

The study population included 16 518 females with a mean follow‐up of 27.9 (SD 7.0) years (Table 1). Follow‐up was for the period between 1993 and 2021. The mean age at baseline was 54.8 (SD 3.8, range 46–61). The all‐cause mortality during the follow‐up was 36.8% (*n* = 6076). Altogether, 7.8% of women (*n* = 1308) were diagnosed with AMD during the follow‐up. The mean age at diagnosis of dAMD was 76.9 years (SD 6.1, *n* = 847, 64.7% of all AMD diagnoses) and 78.1 years (SD 5.7, *n* = 461, 35.2% of all AMD diagnoses) at the diagnosis of nAMD. Later in the results label ‘AMD’ includes all AMD patients (both dAMD and nAMD).

**TABLE 1:** Characteristics of the study population, with categories of interest for long‐term diabetes medication, antithrombotic medications, corticosteroids or immunosuppressants.

	*n*	Body mass index (SD)	Mean treatment duration, years
Study population	16 518	27.0 (4.2)	N/A
Diabetes	1988	29.6 (4.7)	13.6 (8.1)
Antithrombotic	8027	27.5 (4.2)	9.3 (6.9)
Corticosteroids	5751	27.6 (4.3)	9.2 (8.3)
Immunosuppressants	796	26.9 (4.0)	7.7 (6.7)

Abbreviations: N/A, not applicable; SD, standard deviation.

Patients with diabetes medication in use (*n* = 1988) had a mean of 12.8 (SD 8.5) years of medication history during the follow‐up (Table 1). Out of these, during the follow‐up 950 patients had diabetes medication without metformin and 1038 patients with metformin or combination treatment, including metformin. Altogether, 1501 patients had over 5 years of medication and were considered long‐term users in the analysis.

All patients with anti‐thrombotic medication (*n* = 8027) had a mean of 8.4 (SD 7.1) years of medication history during the follow‐up, including 5596 patients with over 5 years of medication, considered long‐term users. (Table 1). Among AMD patients, during the follow‐up 626 patients had long‐term antithrombotic medication, whereas 682 did not. Among all women with long‐term antithrombotic medication, 2039 were vitamin K antagonist users, 1801 patients whose medication belonged to the heparin group, 4518 used platelet aggregation inhibitors excluding heparin, 85 used direct thrombin inhibitors, 410 used direct factor Xa inhibitors, and 198 used other antithrombotic agents. Altogether, patients with any use of systemic corticosteroids (*n* = 5751) had a mean of 6.6 (SD 8.2) years of medication history during the follow‐up, including 2382 long‐term users (Table 1). During the follow‐up patients with immunosuppressants (*n* = 796) had a mean of 7.1 (SD 6.8) years of medication history (Table 1). Out of these, 407 are long‐term users. Some overlap between categories of long‐term medication usage was present in the cohort. The proportion of women with simultaneous long‐term use in two, three or four categories of medications was *n* = 1970 (11.8%), *n* = 300 (1.8%) and *n* = 14 (0.1%), respectively.

### 3.2 Age at diagnosis and risk for AMD

In the study population, the cumulative prevalence of all AMD during the follow‐up with and without long‐term diabetes medication is described in Table 2 (Pearson chi‐squared test). Overall, diabetes medications did not affect the mean age at the time of AMD diagnosis or cumulative prevalence of AMD. However, a significant difference in AMD diagnosis age was observed when long‐term non‐metformin users were compared to the diabetes control group (*p* = 0.026). Regardless of the numerical difference, the results were not significant when long‐term metformin users and long‐term non‐metformin users were compared: the mean AMD age was 78.3 (6.1) and 75.3 (5.4) years, respectively (*p* = 0.50). Prevalence of AMD decreased among women without long‐term anti‐thrombotic medication compared to the group who had long‐term use of antithrombotic medication (*p* < 0.001). Age at AMD was lower among women without long‐term antithrombotic medication than in the group of long‐term users of antithrombotic medication (*p* < 0.001). Prevalence of AMD was lower among women without long‐term systemic corticosteroid use compared with persons who had long‐term use of systemic corticosteroids (*p* < 0.001). However, we did not observe changes at the mean age of AMD diagnosis between non‐users or long‐term users of corticosteroids. Prevalence of AMD was lower among women without long‐term immunosuppressant use than in the group of who had long‐term use of immunosuppressants (*p* < 0.001). We did not observe changes in the mean age at AMD between the immunosuppressant control group and long‐term users of immunosuppressants. Control groups (women without long‐term medication) contained both non‐users and women who had used specific medication under 5 years before the AMD diagnosis. The age at AMD diagnosis analysis, where non‐users and persons with <5 years of medication history are separated, can be found in Supplemental Table 1.

**TABLE 2:** Prevalence of AMD, age‐adjusted hazard ratio, and mean age at diagnosis among long‐term medication users (use >5 years) and their respective controls (no use or use <5 years).

	AMD prevalence, % (95% CI) during follow‐up	HR (*p* vs. control)	Mean age at AMD (years, SD)	*p* (age vs. control)
Study population	7.8 (7.4–8.2)		77.4 (6.0)	
Diabetes				
Medication (any)	9.0 (7.7–10.3)	1.075 (0.488)	77.1 (6.0)	0.754
Metformin	9.2 (7.7–10.3)	0.879 (0.401)	78.4 (6.1)	0.293
Other that metformin	8.7 (6.9–10.5)	1.107 (0.518)	75.3 (5.4)	**0.026**
Control	7.8 (7.4–8.2)		77.3 (6.0)	
Antithrombotic				
Medication	11.2 (10.5–11.9)	1.145 **(0.042)**	77.9 (5.7)	**<0.001**
Control	6.2 (5.7–6.7)		76.1 (6.4)	
Corticosteroids				
Medication	11.5 (10.7–12.3)	1.348 **(<0.001)**	77.4 (5.9)	0.994
Control	7.3 (6.8–7.8)		77.4 (6.0)	
Immunosuppressants				
Medication	13.8 (11.3–16.1)	1.623 **(0.004)**	77.5 (5.4)	0.875
Control	7.7 (7.3–8.1)		77.3 (6.0)	

*Note*: Pearson chi‐squared and *t*‐tests were used for statistical analysis. Bold indicates Significant values.

Abbreviations: AMD, age‐related macular degeneration; HR, hazard ratio; SD, standard deviation.

### 3.3 5‐Year incidence of AMD in age matched groups

We further analysed the incidence of AMD during the 5 years period in age matched groups. Age matched groups were created for ages 65, 70, 75, 80 and 85. When age‐matched groups were compared for AMD incidence, no significant differences were observed between the diabetes control group and long‐term diabetes medication users (Table 3). When the antithrombotic control group was compared to the long‐term antithrombotic users, a difference was only observed in the age category between 65 and 70 years (Table 3). In age category between 65 and 70 years, 0.63% (96 AMD cases out of 15 320 persons) of women in the antithrombotic control group and 1.46% (7 AMD cases out of 478 persons) in the antithrombotic long‐term exposure group developed AMD during the 5‐year observation period (*p* = 0.024). When age‐matched groups were compared for AMD incidence among systemic corticosteroid users, significant differences were observed in age category between 65–70 and 80–85 years (Table 3). In age category between 65 and 70 years, 0.6% (93 AMD cases out of 15 092 persons) of women in the control group for systemic corticosteroids and 1.4% (10 AMD cases out of 706 persons) in the systemic corticosteroid long‐term exposure group developed AMD during the 5‐year observation period (*p* = 0.009). In age category between 80 and 85 years, 3.5% (300 AMD cases out of 8569 persons) of women in the systemic corticosteroid control group and 4.6% (72 AMD cases out of 1555 persons) in the systemic corticosteroid long‐term exposure group developed AMD during the 5‐year observation period (*p* = 0.010). Significant differences were observed in age category between 65–70 and 80–85 years, when AMD incidence was compared between the immunosuppressant control group and immunosuppressant long‐term users (Table 3). In age category between 65 and 70 years, 0.6% (101 AMD cases out of 15 755 persons) of women in the immunosuppressant control group and 4.8% (2 AMD cases out of 42 persons) in the immunosuppressant long‐term exposure group developed AMD during the 5‐year observation period (*p* = 0.001). In age category between 80 and 85 years, 3.6% (357 AMD cases out of 9888 persons) of women in the immunosuppressant control group and 6.4% (15 AMD cases out of 236 persons) in the immunosuppressant long‐term exposure group developed AMD during the 5‐year observation period (*p* = 0.004).

**TABLE 3:** AMD incidence in age‐matched cohorts with 5‐year increments. The control group includes women with no long‐term medication at the age of interest.

	Age matching group, years	AMD incidence during next 5 years: control group vs. long‐term, %	*p*
Diabetes	65	0.7 vs. 0.7	0.926
	70	1.6 vs. 2.4	0.076
	75	3.0 vs. 3.8	0.359
	80	3.7 vs. 3.3	0.687
	85	2.9 vs. 3.0	0.772
Antithrombotic	65	0.6 vs. 1.5	**0.024**
	70	1.7 vs. 1.6	0.976
	75	2.9 vs. 3.8	0.140
	80	3.6 vs. 3.8	0.240
	85	3.0 vs. 2.7	0.793
Corticosteroids	65	0.6 vs. 1.4	**0.009**
	70	1.2 vs. 2.2	0.076
	75	3.0 vs. 3.9	0.155
	80	3.5 vs. 4.6	**0.010**
	85	2.8 vs. 3.5	0.201
Immunosuppressants	65	0.6 vs. 4.8	**< 0.001**
	70	1.6 vs. 2.5	0.398
	75	3.0 vs. 5.2	0.107
	80	3.6 vs. 6.4	**0.004**
	85	2.9 vs. 2.4	0.889

*Note*: Bold indicates Significant values.

Abbreviation: AMD, age‐related macular degeneration.

## 4 DISCUSSION

Increasing evidence suggests that both local changes in the retinal microenvironment and systemic components are involved in the development of AMD. Recent observations of metformin, anticoagulants and factors related to inflammation in AMD have been under active discussion (Aggarwal et al., 2024; Jiang et al., 2022; Xu et al., 2021; Yan et al., 2022; Yang et al., 2024). Thus, we explored long‐term exposure to systemic medications, including diabetes medications, antithrombotics, corticosteroids and immunosuppressants, with the incidence of AMD. More specifically, we focused on the odds of AMD development and long‐term use of the medications for over 5 years. Long‐term use of medication was collected based on reimbursed medication purchases.

Since there is a substantial amount of evidence that the low‐level inflammation involves AMD pathogenesis, we anticipated whether systemic use of anti‐inflammatory agents is associated with AMD (Helotera & Kaarniranta, 2022; Khan et al., 2022; Nahavandipour et al., 2020). Interestingly, corticosteroid and immunosuppressant users were enriched among AMD patients, but age at diagnosis was not affected. Based on available information, it would have been expected that lowering serum levels of IL‐6 and TNF‐α would have decreased the odds of nAMD or delayed diagnosis (Khan et al., 2022; Nahavandipour et al., 2020). However, increased odds may reflect more the underlying active and treatment‐resistant disease that secondarily increase the odds of AMD. It was recently reported that lupus erythematosus, Sjogren's syndrome, giant cell arteritis, and Crohn's autoimmune diseases show an association with increased odds of AMD (Moir et al., 2023). Interestingly, when 5‐year AMD incidence was analysed in age‐matched groups, both corticosteroid and immunosuppressant users had higher incidences of AMD than their comparators in 2 out of 5 analysed age periods. In most of the other age‐matched corticosteroid and immunosuppressant long‐term use groups, a similar trend was observed. A higher incidence of AMD among corticosteroid and immunosuppressant long‐term users, both in total population and in age‐matched groups, was observed. We consider that the role of underlying inflammatory diseases during AMD progression should be examined in more detail in the future. However, we consider that patient numbers in some of the subgroups in 5‐year incidence analysis were too low to make strong conclusions, and observed differences should be confirmed with the bigger patient population.

Metformin targets multiple pathways of ageing and thus use of metformin as a treatment of age‐related diseases such as AMD is of interest (Barzilai et al., 2016). Recently, Aggarwal et al. reported that use of metformin in non‐diabetic patients can reduce the odds of AMD development (Aggarwal et al., 2024). Instead, inconsistent results from the protective association have been received from patients with diabetes (Gokhale et al., 2023; Lee et al., 2019; Romdhoniyyah et al., 2021). In our data set, metformin long‐term users showed a trend towards later diagnosis for AMD and decreased odds for AMD development than their comparators. However, these results were not statistically significant, which may reflect the limited number of patients. As no statistical significance was observed for metformin in basic analyses, diabetes medication long‐term users were overseen only as one group during age matched analyses for AMD incidence. In general, long‐term use of diabetes medication does not seem to affect the prevalence and incidence of AMD in our data set. However, when long‐term metformin users were separated from diabetes medication long‐term users, an earlier diagnosis age for AMD was observed among patients who did not receive metformin.

It has been shown previously that anticoagulant dabigatran may reduce risk for new nAMD (Akter et al., 2022). Akter et al. further showed that thrombin reduces transepithelial resistance of RPE cells, generates complement component C3 and C5 cleavage products, leads to C3d/membrane attack complex deposition on cell surfaces and increased connective tissue growth factor expression and VEGF secretion (Akter et al., 2022). As anticoagulants are thrombin inhibitors, interfering with thrombin levels or mechanism of action, one could hypothesize that anticoagulants would increase blood–retina barrier integrity and reduce pressure for VEGF‐driven neovascularization. In line with Akter et al.'s findings, we recently showed from the smaller patient population that patients on antiaggregation (ASA or clopidogrel) and anticoagulation (warfarin or direct oral anticoagulants, DOACs) medication receive nAMD diagnosis later than their comparators. Difference was especially noticeable among anti‐coagulation medication users and patients with anticoagulation medication also stayed longer on anti‐VEGF treatment than patients without anti‐coagulation medication (Helotera et al., 2024). Based on ours and Akter's previous findings, especially role of DOACs during nAMD progression would be interest to explore more in detail. Unfortunately, we could not conduct a separate analysis of either nAMD or dAMD patients among DOAC users from total antithrombotic medication due to the low number of cases in our study (data not shown). Thus, in this study, we performed our analyses only for the whole AMD population and the whole anti‐thrombotic group. We observed that patients with antithrombotic medication were diagnosed with nAMD later than their comparators. However, at the same time, it was observed that prevalence of AMD was enriched among patients with antithrombotic medication This enrichment may be more related to the underlying cardiovascular disease than to the antithrombotic medication itself. When the 5‐year incidence of AMD was analysed from age‐matched groups, anti‐thrombotic medication users showed increased incidence of AMD in 65 years analysis, but in the other four analyses, no significant difference was observed, and no trend was observed. As we could not separate nAMD patients and DOAC users, the question, whether delayed onset AMD among DOAC users is because these patients are just older when initiating the treatment or if the medication modulates the nAMD disease progression could not be answered.

In our current analysis, one limiting factor is that the cohort was still relatively young for an AMD study, and it is expected that more AMD cases will arise in the future. For example, in our recent study median age for treatment initiation for nAMD was 79 years, reflecting also the age at diagnosis (Helotera et al., 2024). Thus, while the study population ages, we consider that analyses for 5‐year incidences, comparison of different anti‐thrombotic medications as well as diabetes medications should be revisited. Moreover, our analysis focused only on reimbursed medication purchases, and medications given in hospitals are not included in this analysis. Thus, future analysis would also benefit from including medication provided in hospitals.

In this study, we could not differentiate whether observed differences occur due to medications used or underlying baseline disease. We consider that both options have potential to affect AMD progression. For example, low‐level inflammation is known to be linked to AMD development and thus systemic changes, for example, generalized low‐level inflammation due to baseline disease has potential to drive AMD progression. For example, by current knowledge, some of the autoimmune diseases including Crohn's disease and systemic lupus erythematosus may be associated with modest increase in the odds for AMD (Moir et al., 2023). Based on our results, systemic changes of the most common baseline diseases behind the use of systemic corticosteroid and immunosuppressant, for example, asthma, polymyalgia rheumatica and rheumatoid arthritis, and their role for AMD development should be examined in more detail in the future.

Moreover, our study spans 1993–2021 and the diagnosis and treatment of AMD have evolved during this period. We consider that the emergence of anti‐VEGF treatments for nAMD 20 years ago may have affected the recording of the nAMD cases (Bege et al., 2025). Approval of anti‐VEGF treatments led also to the utilization of OCT in the diagnosis of AMD. Before the introduction of OCT, fluorescence angiography was used for diagnosis. We believe that the use of OCT has increased the accuracy of diagnoses. However, AMD is most often diagnosed in the elderly population and as the mean age at baseline in our study was 54.8 (SD 3.8), most of the nAMD diagnoses in the study population were expected to rise when anti‐VEGF treatments were already available. Though early cases of AMD may not be captured in patient records due to the lack of national screening programs and treatment options. Thus, numbers presented in this study may underestimate the incidence and prevalence of AMD.

This study shows that people with long‐term corticosteroids and immunosuppressants are at greater risk for developing AMD. Also, women with antithrombotic medication showed increased odds for AMD throughout the entire follow‐up, but 5‐year AMD incidence analysis for age‐matched groups did not support this finding as strongly as it did with the corticosteroids and immunosuppressants. Interestingly, the antithrombotic medication users were diagnosed later for AMD than their comparators.

## CONFLICT OF INTEREST STATEMENT

The authors report no conflicts of interest.

## Supporting information


Data S1.
