# Supplementary material for: The impact of systemic long‐term medications for the development of age‐related macular degeneration
Source: Acta Ophthalmol. 2026 Mar 6;104(6):674–81. doi: 10.1111/aos.70113 (PMC13460348; doi:10.1111/aos.70113)
Supplement: Supplementary file 1 [file AOS-104-674-s001.docx]

Supplemental table 1. Age at diagnosis of non-users, short term users and long-term users. Abbreviations: AMD, age-related macular degeneration; SD, standard deviation

|  | **Category of use** | **n, AMD / total (AMD prevalence, %)** | **Mean age at AMD, (SD)** | **p-value* (AMD age)** |
| --- | --- | --- | --- | --- |
| **Diabetes medication** | Non-user | 1160 / 14681 (7.9) | 77.4 | 0.754 |
|  | Short-term in use, <5 years | 14 / 336 (4.1) | 76.5 |  |
|  | Long-term use, ≥ 5 years | 135 / 1501 (9.0) | 77.1 |  |
| **Antithrombotic medication** | Non-user | 489 / 8491 (5.8) | 76.4 | < 0.001 |
|  | Short-term in use, <5 years | 193 / 2431 (7.9) | 77.8 |  |
|  | Long-term use, ≥ 5 years | 627 / 5596 (11.2) | 77.9 |  |
| **Systemic corticosteroids** | Non-user | 887 / 12474 (7.1) | 77.4 | 0.599 |
|  | Short-term in use, <5 years | 147 / 1662 (8.8) | 76.9 |  |
|  | Long-term use, ≥ 5 years | 275 / 2382 (11.5) | 77.4 |  |
| **Immunosuppressants** | Non-user | 1227 / 16002 (7.7) | 77.3 | 0.543 |
|  | Short-term in use, <5 years | 26 / 317 (8.2) | 78.6 |  |
|  | Long-term use, ≥ 5 years | 56 / 407 (13.8) | 77.5 |  |
| * ANOVA Tukey’s test between categories | | | | |
